# 1D *vs.* 2D shape selectivity in the crystallization-driven self-assembly of polylactide block copolymers†
†Electronic supplementary information (ESI) available: Further polymer and nanostructure characterisation. See DOI: 10.1039/c7sc00641a



**DOI:** 10.1039/c7sc00641a

**Published:** 2017-04-13

**Authors:** Maria Inam, Graeme Cambridge, Anaïs Pitto-Barry, Zachary P. L. Laker, Neil R. Wilson, Robert T. Mathers, Andrew P. Dove, Rachel K. O'Reilly

**Affiliations:** a Department of Chemistry , University of Warwick , Gibbet Hill , Coventry , CV4 7AL , UK . Email: a.p.dove@warwick.ac.uk ; Email: r.k.o-reilly@warwick.ac.uk; b Department of Physics , University of Warwick , Gibbet Hill , Coventry , CV4 7AL , UK; c Department of Chemistry , Pennsylvania State University , New Kensington , Pennsylvania 15068 , USA

## Abstract

2D materials such as graphene, LAPONITE® clays or molybdenum disulfide nanosheets are of extremely high interest to the materials community as a result of their high surface area and controllable surface properties. While several methods to access 2D inorganic materials are known, the investigation of 2D organic nanomaterials is less well developed on account of the lack of ready synthetic accessibility. Crystallization-driven self-assembly (CDSA) has become a powerful method to access a wide range of complex but precisely-defined nanostructures. The preparation of 2D structures, however, particularly those aimed towards biomedical applications, is limited, with few offering biocompatible and biodegradable characteristics as well as control over self-assembly in two dimensions. Herein, in contrast to conventional self-assembly rules, we show that the solubility of polylactide (PLLA)-based amphiphiles in alcohols results in unprecedented shape selectivity based on unimer solubility. We use log *P*_oct_ analysis to drive solvent selection for the formation of large uniform 2D diamond-shaped platelets, up to several microns in size, using long, soluble coronal blocks. By contrast, less soluble PLLA-containing block copolymers yield cylindrical micelles and mixed morphologies. The methods developed in this work provide a simple and consistently reproducible protocol for the preparation of well-defined 2D organic nanomaterials, whose size and morphology are expected to facilitate potential applications in drug delivery, tissue engineering and in nanocomposites.

## Abstract 2

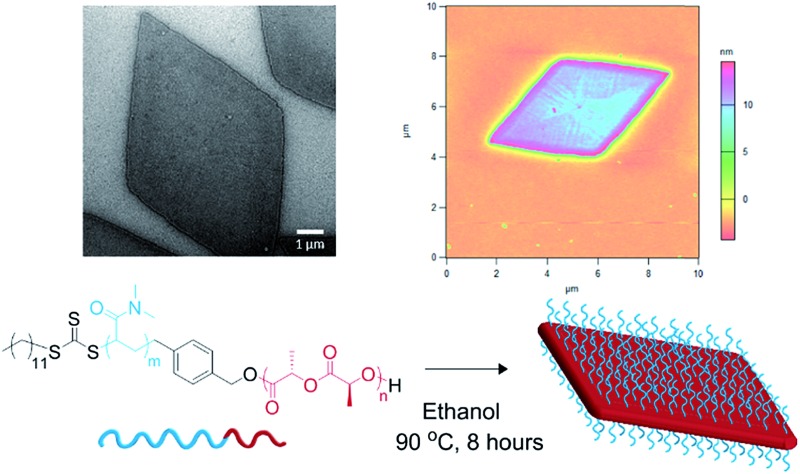
Assemblies of polylactide-based amphiphiles in alcohols are shown to give unprecedented shape selectivity based on unimer solubility, leading to the formation of large uniform 2D diamond-shaped platelets, up to several microns in size.

## Introduction

Conventional solution self-assembly occurs when a block copolymer is dissolved in a solvent that is selective for one of the blocks or occurs during polymerization in a selective solvent for one of the blocks.^1–3^ Self-assembly is driven by a balancing of energies associated with solvation of the corona and chain packing of the core block and their relative ratio often determines the resultant micellar morphology.^3^ A wide range of morphologies are accessible using this methodology, however access to free-standing sheet formation (*i.e.* 2D materials with a high aspect ratio) is often challenging, with limited examples in the literature,^4–7^ due to the prevalence of the formation of closed structures such as vesicles and cylinders. Yet, free-standing sheet formation is often seen in inorganic materials assemblies such as nanosheets of molybdenum disulfide, boron nitride and LAPONITE® clays. Indeed, the discovery of graphene as a 2D material analogy of 1D carbon nanotubes has provided unheralded interest from the materials community. Such 2D high aspect materials are important as additives in composites,^8–10^ thermosets^11^ and as a platform for nanoparticles.^12–15^

Crystallization-driven self-assembly (CDSA) is a novel tool in the solution polymer self-assembly toolbox and has been utilized to create an impressive range of hierarchical block copolymer structures.^16^ Unlike in conventional solution self-assembly, where the range of morphologies obtained are determined by varying the relative block composition of each block, polymers assembled *via* CDSA favor the formation of micelles with low interfacial curvature. Winnik and Manners have utilized the CDSA of poly(ferrocenyldimethylsilane) (PFS) block copolymers for the preparation of a wide range of high aspect nanostructures including cylinders^17–20^ and platelet micelles.^21–24^ However, despite these advances there are relatively few examples where the aggregate morphology can be readily controlled to form nanostructures whose size can be controlled in two dimensions.^14,25–30^ Indeed, this was reported by Winnik and Manners through the utilization of CDSA to afford 2D platelet assemblies, which could be extended to grow in 2D to form micron-sized lenticular micelles of complex function and form.^31^ In these studies, it was shown that lamellae/platelets were obtained for block copolymers that have equivalent corona–core degrees of polymerization, while an increase in the degree of polymerization of the corona-forming block led to cylindrical morphologies.^24^ This phenomenon was observed even when the corona-forming block was much larger than the core-forming block (20 : 1 block ratio).^32^ A further report by Chen and coworkers, utilized similar block ratios with a poly(ε-caprolactone) crystalline segment to afford elongated polymer platelets with hexagonal edges.^14^

The only other report of the formation of such high aspect ratio nanostructures using CDSA was by Eisenberg, who highlighted the utilization of CDSA and homopolymer co-assembly techniques (based on a poly(ε-caprolactone) core-forming block) to allow for the formation of 2D block copolymer ‘rafts’.^33,34^ This approach utilized the hierarchical growth of lamellae from one dimensional rods and demonstrated the first example of the formation of highly elongated subunits (aspect ratio > 50) through spontaneous alignment without the presence of a foreign interface. This evolution of dimensionality from 1D to 2D structures was attributed to the added PCL homopolymer which was acting as a structure-driving agent.

Our group has pioneered research in the area of CDSA of amphiphilic poly(l-lactide) (PLLA)-based block copolymers.^35–37^ PLLA is a biocompatible semi-crystalline polymer as well as being derived from renewable resources and has found extensive use in delivery applications.^38^ Previously, we have shown that CDSA is possible for various PLLA-containing block copolymers such as *N*,*N*-dimethylacrylamide, ethylene glycol or 4-acryloyl morpholine.^39^ To date, we have focused on the self-assembly of polyacrylic acid containing copolymers, PAA-*b*-PLLA, polymerized *via* ring opening polymerization (ROP) and reversible addition–fragmentation chain transfer (RAFT) polymerization, where cylindrical morphologies have been obtained with varying block compositions.^39,40^ It is clear however, that CDSA rules cannot be easily generalized and translated between different polymers, and hence requires optimization of solvent systems and assembly conditions to promote the process efficiently for each system.

There is also interest in using CDSA to develop fully biocompatible and degradable high aspect ratio nanostructures for utilization in nanomedicine applications.^41^ For example, Chen and coworkers showed that poly(ethylene oxide)-*b*-poly(ε-caprolactone) leaf-like sheets showed a selective internalization to different cells.^27^ A number of reports also indicate that elongated morphologies clearly outperform their spherical analogues in terms of escape from phagocytosis and firm binding to the target tissue.^42,43^ For example, DeSimone used a series of nanoparticles of the same shape but with differing aspect ratios to demonstrate (using particle replication in non-wetting templates technique) different levels of cellular uptake; specifically, those of higher aspect ratio showed faster uptake kinetics.^44^ Indeed, it has been reported that particle shape (specifically the local particle shape at the point of initial contact) and not size plays a dominant role in phagocytosis and intracellular transport.^45–47^

In this work we use, for the first time, polymer hydrophobicity calculations from log *P*_oct_ analysis techniques to direct the formation of 2D nanostructures *via* CDSA in a single component solution-phase protocol. In sharp contrast to previous reports, platelets were observed for block copolymers with large corona–core block ratios (without the presence of homopolymers), while cylindrical structures were observed for smaller corona–core block ratios. We have also been able to demonstrate a novel blending methodology to allow for access to more complex 2D nanostructures. This methodology provides hitherto unprecedented access to well-defined 2D organic nanomaterials, which are difficult to access using traditional assembly methods and are expected to have potential as biocompatible nanomaterials for application as components in biomaterials and/or delivery applications.

## Results and discussion

**Scheme 1:**
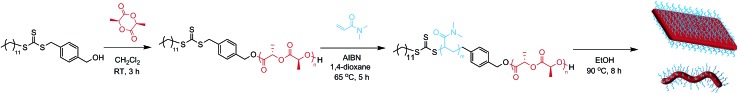
Synthesis of PDMA-*b*-PLLA cylinders and diamond-shaped platelets.

Diblock copolymers were synthesized using a previously reported method (Scheme 1, Table 1).^35^ ROP of l-lactide yielded a PLLA macroinitiator, and subsequent RAFT polymerization of *N*,*N*-dimethylacrylamide (DMA) was used to prepare the corona block. SEC analysis revealed monomodal polymers with relatively low dispersities (*Đ*_M_) and the absence of PLLA homopolymer as confirmed by DOSY NMR analysis (Fig. S1 and S2†).

**Table 1:** Characterization of block copolymers PDMA_*n*_-*b*-PLLA_*m*_

	*M*_n_ ^*a*^ (kg mol^–1^)	*Đ*_M_ ^*a*^	*m* : *n* ^*b*^	Hydrophobic wt%^*c*^
PDMA_1000_-*b*-PLLA_48_	122.2	1.10	20 : 1	6.9
PDMA_600_-*b*-PLLA_48_	74.1	1.06	12.5 : 1	11.0
PDMA_250_-*b*-PLLA_48_	41.5	1.05	5 : 1	22.8
PDMA_150_-*b*-PLLA_48_	28.2	1.05	3 : 1	33.0
PDMA_250_-*b*-PLLA_25_	36.3	1.17	10 : 1	13.9
PDMA_130_-*b*-PLLA_25_	25.0	1.10	5 : 1	23.7

^*a*^Apparent values based on SEC measurements.

^*b*^Ratio of degrees of polymerization calculated from ^1^H NMR integration.

^*c*^Weight percentages calculated from ^1^H NMR integration. Note that all PLLA wt% values lie within the previously identified region to undergo CDSA processes which yield cylindrical micelles.^40^

### Directing self-assembly conditions using log *P*_oct_ analysis

**Fig. 1:**
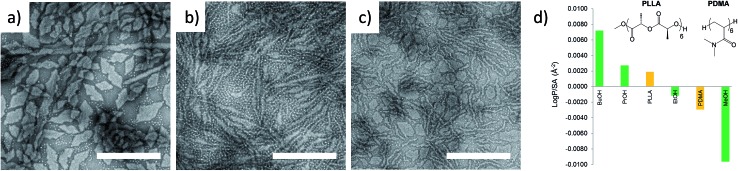
TEM micrographs of corona–core ratio 12.5 : 1 PDMA_600_-*b*-PLLA_48_ self-assembled in (a) ethanol, (b) *n*-propanol and (c) *n*-butanol at 65 °C for 18 h and cooled to room temperature. All samples were stained with uranyl acetate. Scale bar = 1 μm. (d) Structure of hexameric models based on polylactide (PLLA) and poly(*N*,*N*-dimethylacrylamide) (PDMA) and log *P*_oct_ hydrophobicity calculations compared to methanol (MeOH), ethanol (EtOH), propanol (PrOH), and butanol (BuOH). A similar trend was noted with oligomeric models composed of octamers. PLLA incorporated a MeO initiator with an OH endgroup. PDMA was hydrogen terminated (see Fig. S3†).

In order to select the most appropriate solvent for self-assembly, we investigated the effects of polymer solubility on nanostructure formation, where we sought to define a single, alcoholic solvent that could be selective for the corona block. As such, a series of molecular hexameric models of PLLA and PDMA were constructed, where the average amount of hydrophobicity was determined for each block and compared to the hydrophobicity of various alcoholic solvents. To quantify hydrophobicity, octanol–water partition coefficients (log *P*_oct_) were calculated and normalized by surface area (SA) (Fig. 1). Previously, log *P*_oct_ values have provided a convenient method to quantify the hydrophobicity of monomers,^48^ homopolymers and copolymers,^49^ and crosslinked networks.^50^ As such, we theorized that they could also be used to provide a simple and reliable tool for the prediction of solvents for block copolymer self-assembly based on solubility. Compared to assessing hydrophobicity with Hildebrand solubility parameters, log *P*_oct_/SA values enable faster assessment time and provide a physical meaning that can be experimentally verified. For instance, log *P*_oct_/SA values for homopolymers and copolymers correlate to contact angle measurements, swelling experiments, and Nile red absorbances.^51^ These calculations demonstrate that the hydrophobicity of the polymer can be correlated to the optimal hydrophobicity of the solvent in order to promote unimer solubility and allow access to well-defined constructs. Interestingly, the calculated log *P*_oct_ values revealed that ethanol more closely resembled the hydrophobicity of PDMA compared to *n*-propanol, *n*-butanol, and methanol. This is in contrast to predictions made using the Hildebrand system, where the solubility parameters of PDMA (25.4) and the alcohols used (ethanol (26.5), *n*-propanol (24.6), and *n*-butanol (23.2))^52^ predict that *n*-propanol would be the optimum solvent for PDMA.

The results from the log *P*_oct_ analysis of our polymers were initially tested by investigating the self-assembly of PDMA_600_-*b*-PLLA_48_ (block ratio 12.5 : 1) in a range of alcoholic solvents. Assembly was performed in ethanol, *n*-propanol and *n*-butanol at 65 °C for 18 h followed by slow cooling to room temperature (analogous to the conditions used in our previous CDSA studies).^35^ Consistent with log *P*_oct_ analysis, TEM imaging revealed that more well-defined 2D platelets and faceted lamellae were obtained from ethanol, whereas elongated or ill-defined structures were observed in *n*-propanol and *n*-butanol (Fig. 1). This confirmed ethanol as the optimum solvent for use in further investigations of well-defined 2D nanostructures and highlights the potential utility of log *P*_oct_ as an indicator of solubility parameters for self-assembly.

### Optimizing the conditions for self-assembly

**Fig. 2:**
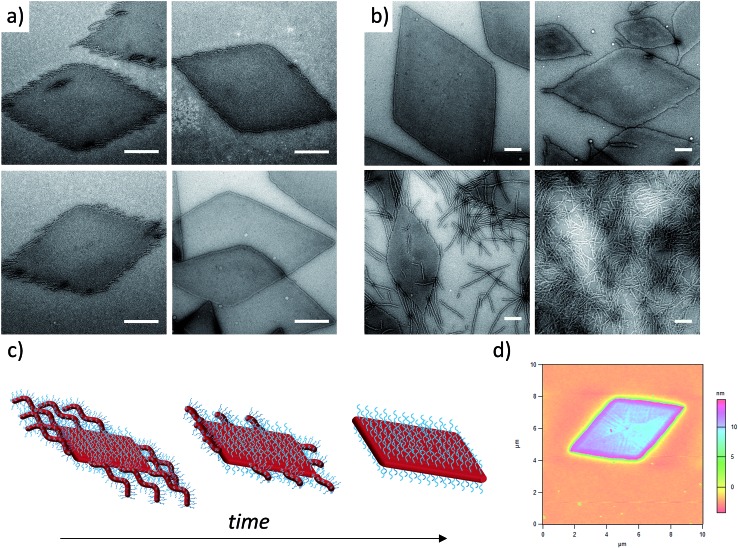
(a) TEM micrographs of corona–core ratio 20 : 1 PDMA_1000_-*b*-PLLA_48_ self-assembled in ethanol at 90 °C for 2 h (top left), 4 h (top right), 6 h (bottom left) and 8 h (bottom right) before cooling to room temperature. (b) TEM micrographs of a series of PDMA_*m*_-*b*-PLLA_48_ block copolymers of corona–core ratios of 20 : 1 (*m* = 1000, top left), 12.5 : 1 (*m* = 600, top right), 5 : 1 (*m* = 250, bottom left), and 3 : 1 (*m* = 150, bottom right). Samples were self-assembled in ethanol at 90 °C for 8 h and cooled to room temperature. All samples were stained with uranyl acetate. Scale bar = 1 μm. (c) Schematic of diamond platelet formation kinetics. (d) AFM of diamond platelet assembled from 20 : 1 corona–core ratio diblock copolymer.

In order to enhance 2D particle formation, we theorised that increasing unimer solubility would reduce the dispersity of the assemblies. Our initial investigations into alternative solvents using log *P*_oct_ analysis demonstrated a poorer solubility with *n*-propanol and *n*-butanol, as shown previously, and methanol was found to fully solubilise the unimers (thus no structures were formed). Hence, we investigated the effect of elevated temperature and prolonged heating in ethanol to increase the solubility of the unimers prior to assembly. Indeed, at longer heating times, kinetic studies at 90 °C revealed increasingly well-defined diamond platelets (Fig. 2a) for the largest corona–core ratio (20 : 1) of up to 10 μm in length and *ca.* 15 nm thick (Fig. 2d and S4†), where 8 h was determined to be the optimum time required to achieve consistently reproducible smooth diamond-shaped platelets. These structures are similar to those observed for PLLA single crystals where “lozenge” shaped crystals are reported.^53,54^ The concentration dependent assembly of the diamond platelets showed no discernible change in morphology, particle dispersity or size at concentrations up to 25 mg mL^–1^. Notably, all of the observed diamonds were consistently larger than those formed at 65 °C due to the elevated dissolution temperatures reducing the number of crystalline nuclei, thus producing a smaller number of larger structures. Indeed, extending the heating time further resulted in more platelet structures, even for the smaller corona–core ratios (Fig. S5†).

### Exploring the effect of polymer composition on self-assembly

To further expand the scope of our investigation and determine how the solubility of the coronal block in ethanol affects the self-assembly process, a range of PDMA : PLLA block ratios were synthesized (Table 1). Decreasing the PDMA block length to give 12.5 : 1 and 5 : 1 block ratios (using a PLLA_48_ core block) resulted in mixed phases of structures primarily diamond in shape, with clear dispersity in size, and evidence of elongated ends and cylindrical micelles (Fig. 2b). In comparison, purely cylindrical structures were obtained from the lowest PDMA block length (3 : 1), which suggests that under these conditions, the crossover composition^31^ has been reached in this system. Similar observations (Fig. S6†) were made during the assembly of a second PLLA block which had a lower DP (PDMA_250_-*b*-PLLA_25_) but was more similar to the PLLA block lengths previously reported by our groups (where no evidence of 2D structures was observed).^40^ These observations are in stark contrast to the widely reported PFS system, where elongated structures are formed when the corona-forming block is much larger than the core-forming block^24^ to accommodate the large volume occupied by the corona chains.^55^

### Characterization of the assemblies

To further investigate the dimensions of specific 2D diamond-shaped nanostructures in solution, small angle X-ray scattering (SAXS) analysis was performed.^56^ It has been demonstrated that at intermediate *q* values, the scattering intensity *I*(*q*) is proportional to *q* ^–*D*^ with *D* being the fractal exponent of the scattering objects, where dispersed plate-like objects have a *D* value of 2 while aggregates or folded structures have typical *D* values between 3 and 4.^57^ Upon examination, the 20 : 1 corona–core ratio platelets (Fig. 2a) exhibit a slope of –2 for intermediate *q* values (Fig. S7a†), which confirms the presence of one-dimensional objects as observed by TEM. The early stage of a plateau is observable at low *q* values,^58^ with a repeat distance which correlates closely with the platelet sizes observed by TEM analysis. The slope observed at low *q* values is close to –3, which suggests that some plates may have stacked together during analysis. The Guinier plot (Fig. S7b†) for flat particles allows the determination of the thickness *τ* from the slope *R*_*τ*_^2^ of the linear region with the following equation: *τ* ^2^ = 12 × *R*_*τ*_^2^ with *R*_*τ*_ being the one-dimensional radius of gyration taken from the center of the platelet perpendicular to the face.^59^ A thickness just below 2 nm is found for the 20 : 1 corona–core ratio PDMA_1000_-*b*-PLLA_48_ platelets. It is expected this thickness mainly relates to the crystalline block as the scattering length density contrast between the solvent and the two blocks is much higher for the crystallized polylactide block than for the amorphous and solvated DMA block.

Selected area electron diffraction (SAED) was also performed on the diamond platelets, formed from the 20 : 1 corona–core ratio diblock copolymer, which confirmed the crystalline nature of the diamonds (Fig. S8†) in addition to wide-angle X-ray scattering (WAXS) analysis (Fig. S9†). The SAED patterns are consistent with the orthorhombic unit cell previously reported for PLLA (with reciprocal lattice parameters; α* = 0.935 nm^–1^, β* = 1.626 nm^–1^, γ* = 90°)^44^ and, along with the diamond shape of the platelets, indicate the {110} growth plane. Furthermore, taking into consideration the fiber repeat distance and molecular weights, we propose that the chain-folding occurs at lamellar surfaces of single crystals. The highly crystalline nature of the assembly also suggests the PLLA component crystallizes in a structure essentially identical to that expected for PLLA on its own.^54^ Although various confined crystal micelles in selective solvents for the amorphous block have been investigated extensively, the formation of well-defined block copolymer crystals is rarely reported.^24,26,60–64^

### Understanding the assembly process

**Scheme 2:**
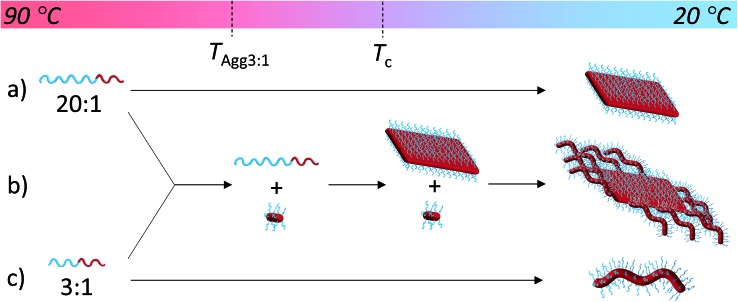
Solvation-driven shape selectivity mechanism (using an arbitrary scale to represent sequential processes on cooling from 90 °C to 20 °C) for PDMA_*m*_-*b*-PLLA_48_ block copolymers of block ratios (a) 20 : 1 (platelet-forming, *m* = 1000); (b) a mixture of 20 : 1 and 3 : 1 and (c) 3 : 1 (cylinder-forming, *m* = 150), where *T*_Agg3:1_ represents aggregation of the 3 : 1 block ratio cylinder-forming block copolymer.

Unlike other systems that utilize CDSA, which rely on solvent quality for the core-forming block,^21^ the formation of these fibers and 2D nanostructures appears to be governed by the interplay between the crystallization of the PLLA core and the solubility of the corona block. On cooling, block copolymers that form unimers above the crystallization temperature of the PLLA block favor crystallization, thus reducing crystal defects and ultimately crystallizing similarly to PLLA homopolymers to form 2D diamond plates (Scheme 2a). In contrast, block copolymers that are less soluble above the crystallization temperature of the PLLA block form aggregates that undergo a crystallization event with epitaxial growth through a unimer exchange process akin to the well-established CDSA principle (Scheme 2c). To provide further evidence for this mechanism, we increased the solubility of the PDMA corona block of a cylinder-forming block copolymer (3 : 1 block ratio) by adding a single acid group to the chain end. Assembly under the same conditions resulted in a change in morphology from pure fibers towards a diamond platelet phase (Fig. S10†).

### Exploring the versatility of this approach

**Fig. 3:**
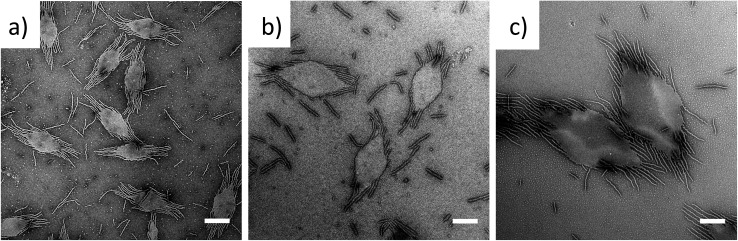
TEM micrographs of PDMA_*m*_-*b*-PLLA_48_ blends of block ratios 20 : 1 (*m* = 1000) and 3 : 1 (*m* = 150), at blending ratios of (a) 25 : 75, (b) 50 : 50 and (c) 75 : 25, self-assembled in ethanol at 90 °C for 8 h and cooled to room temperature. Samples were stained with uranyl acetate. Scale bar = 1 μm.

To exploit this concept in creating complex nanostructures, and inspired by recent work in block copolymer blending,^65–68^ we explored the resultant assembly of mixtures of the two block copolymer compositions (Scheme 2b, Fig. 3). We postulated that blending different ratios of platelet-forming block copolymer (20 : 1 block ratio, PDMA_1000_-*b*-PLLA_48_) and cylinder-forming block copolymer (3 : 1 block ratio, PDMA_150_-*b*-PLLA_48_) and self-assembling in ethanol for 8 h at 90 °C followed by cooling to room temperature, would lead first to assembly of the platelet-forming block copolymers which, in turn, would act as a seed for the aggregated cylinder-forming unimers to undergo epitaxial growth. Satisfyingly, the resultant assemblies did indeed exhibit a diamond center with fibers attached parallel to the long axis of the diamond. While a small number of cylinders are observed in solution (presumably from unavoidable self-nucleation events), fibers on the same side of the diamond grow unidirectionally and are quite uniform in length (Fig. S11†).

## Conclusions

We have demonstrated a simple, single component solution-phase methodology that can be used as an alternative to the commonly applied surface growth approach for block copolymer single crystal preparation, greatly simplifying access to and the design of well-defined 2D organic nanomaterials. It is proposed that these advances will enable this field to fully investigate the potential for these unique and interesting materials towards mimicking the success of their inorganic analogues.

We have further simplified synthetic access to hierarchical nanostructures by demonstrating how log *P*_oct_ analysis can be used to predict optimal solvents for CDSA processes to avoid laborious screening methods in solvent selection, a process that could, with further study, yield significant insights into developing methods to predict solvent systems to direct CDSA. Within this, the importance of solubility in obtaining novel structures has been highlighted, where two factors that influence the solubility of the copolymer were considered; the quality of the solvent for the corona block and the ratio of block lengths. In contrast to previous reports of platelet nanoparticles,^11,69^ diamond-shaped platelets were formed with good solvent quality for the corona block and large corona–core ratios, while more elongated and less defined structures were formed with poorer solvent quality and smaller block ratios. As the polymer becomes less soluble (corona chain length decreases or the solvent quality becomes worse), there is not adequate time for the PLLA chains to adopt a preferred crystal conformation thus resulting in less defined or elongated structures. We propose that the ability of the corona block to solubilize, and thus stabilize, the block copolymer in solution allows the PLLA block to crystallize to a greater extent to yield diamonds which have the appearance of defect-free plates.

Given the high interest in 2D inorganic materials, the ability to readily access and control the assembly of polymers into 2D organic platelets through a simple assembly process provides a platform to develop a range of new materials. Moreover, given their well-defined size, morphology and high stability, applications within nanocomposites, thermosets and platforms for nanoparticle delivery vehicles will be of high interest.
