# Supplementary material for: 1D vs. 2D shape selectivity in the crystallization-driven self-assembly of polylactide block copolymers
Source: Chem Sci. 2017 Apr 13;8(6):4223–30. doi: 10.1039/c7sc00641a (PMC5635812; doi:10.1039/c7sc00641a)
Supplement: Supplementary file 1 [file SC-008-C7SC00641A-s001.pdf]

Electronic Supplementary Material (ESI) for Chemical Science.
This journal is © The Royal Society of Chemistry 2017

Supporting Information for

1D vs 2D shape selectivity in the crystallization-driven self-assembly of polylactide block copolymers

Maria Inam,[a] Graeme Cambridge,[a] Anaïs Pitto-Barry,[a] Zachary Laker,[b] Neil Wilson,[b] Robert T. Mathers,[c] Andrew P. Dove*,[a] and Rachel K. O'Reilly*,[a]

[a]Department of Chemistry, University of Warwick, Gibbet Hill, Coventry, CV4 7AL, United Kingdom
[b]Department of Physics, University of Warwick, Gibbet Hill, Coventry, CV4 7AL, United Kingdom
[c]Department of Chemistry, Pennsylvania State University, New Kensington, Pennsylvania 15068, United States

Materials and Methods

Materials. Chemicals and solvents were purchased from Sigma Aldrich, Acros, Fluka, Fisher Chemical, Alfa Aesar, or VWR. L-Lactide monomer was kindly donated by Corbion-Purac and dried over 3 Å molecular sieves in dichloromethane before recrystallization from toluene and stored in a glovebox with inert atmosphere. 1,4-Dioxane and *N*,*N*-dimethylacrylamide (DMA) were purified by passing through basic alumina before use. (−)-Sparteine was dried over calcium hydride and distilled before use. Bis[(trifluoromethyl)phenyl]-3-cyclohexylthiourea was prepared as previously reported.[1] 2,2'-azobis(2-methylpropionitrile), AIBN, was recrystallized twice from methanol and stored in the dark at 4 °C.

Polymer Synthesis. Poly(*L*-lactide) (PLLA) was synthesized using ring-opening polymerization (ROP) using a functional RAFT agent[2] to yield PLLA macroinitiators of varying DP.[3] Poly(*N,N*-dimethylacrylamide)-*b*-poly(*L*-lactide) (PDMA-*b*-PLLA) was synthesized by chain extending the macro PLLA initiator with *N*,*N*-dimethylacrylamide.[4]

Polymer Characterization. ^{1}H and ^{13}C NMR spectra were recorded at 400 MHz on a Bruker DPX-400 spectrometer in $CDCl_3$ unless otherwise stated. Chemical shifts are reported as δ in parts per million (ppm) downfield from the internal standard trimethylsilane.

Size exclusion chromatography (SEC) measurements in THF were performed on a Varian 390-LC-Multi detector suite fitted with differential refractive index (DRI) and photodiode array (PDA) detectors equipped with a guard column (Varian Polymer Laboratories PLGel 5 µm (50 × 7.5 mm)) and two mixed-D columns (Varian Polymer Laboratories PLGel 5 µm (300 × 7.5 mm)) using THF with 2% triethylamine eluent at a flow rate of 1.0 mL min^{-1}. SEC measurements in DMF were performed on a Varian 390-LC-Multi detector suite system fitted with RI and ultraviolet (UV) detectors (λ = 309 nm) equipped with a PLGel 3 µm (50 × 7.5 mm) guard column and two PLGel 5 µm (300 × 7.5 mm) mixed-D columns using DMF with 0.1% LiBr at 50 °C as the eluent at a flow rate of 1.0 mL min^{-1}. SEC data was calibrated against PS or PMMA standards and analyzed using Cirrus v3.3 software.

Mass spectra were obtained using a Bruker Ultraflex II Matrix-assisted laser desorption/ionisation time of flight (MALDI-ToF) mass spectrometer. Typical preparation of

samples is as follows; *trans*-2-[3-(4-*tert*-butyl-phenyl)-2-methyl-2-propenylidene] malononitrile (DCTB) matrix (20 µL of a 40 mg mL^{-1} HPLC-grade THF solution) was added to the sample (20 µL of a 1 mg mL^{-1} HPLC-grade THF solution) followed by sodium trifluoroacetate (NaTFA) (20 µL of a 0.1 mg mL^{-1} HPLC-grade THF solution) and vortexed before application on a MALDI-ToF plate. Samples were measured in reflectron ion mode and calibrated against SpheriCal (1200 – 8000 g mol^{-1}) standards.

Self-Assembly. As an example of self-assembly conditions, PDMA-*b*-PLLA (10 mg) was added to 2 mL of ethanol (5.0 mg/mL) in a 7 mL vial. The samples were heated in an oil bath at 65 °C or 90 ^{o}C, without stirring for a predetermined period of time before being removed from the oil bath and left to cool to room temperature. Samples were imaged after 1 day of ageing at room temperature.

Transmission Electron Microscopy (TEM). Samples for TEM analysis were prepared by drop casting 7 µL of polymer in ethanol (0.5 mg/mL) onto a carbon/formvar-coated copper grid placed on filter paper. Samples were stained with a 1% uranyl acetate solution to facilitate imaging of the thin organic structures unless specified. Samples were also prepared on graphene oxide support films[5] to negate the necessity for staining. Imaging for samples heated to 65 °C was performed on a Jeol 2000FX transmission electron microscope operating at 200 kV. Imaging for samples heated to 90 °C was performed on a Jeol 2100 transmission electron microscope operating at 120 kV.

Atomic Force Microscopy (AFM). Samples for AFM analysis were prepared by drop casting 7 µL of polymer in ethanol (0.25 mg/mL) onto silicon wafer followed by drying with compressed air. Imaging and analysis were performed on an Asylum Research MFP3D-SA atomic force microscope in alternate contact (tapping) mode.

Selected Area Electron Diffraction (SAED). Samples for SAED analysis were prepared by drop casting 7 µL of polymer in ethanol (0.5 mg/mL) onto a graphene oxide-coated holey Quantifoil grid placed on filter paper. Low-dose SAED was performed on a Jeol 2100 LaB6 transmission electron microscope operating at 200 kV. The diffraction spots from the graphene oxide allow direct and accurate calibration of the diffraction pattern.

Wide Angle X-ray Scattering (WAXS). WAXS was performed on a Panalytical X'Pert Pro MPD equipped with a Cu $K\alpha_1$ hybrid monochromator as the incident beam optics. Typically, *ca.* 30 mg of freeze-dried particles was placed in a 10 mm sample holder, and standard "powder" 2θ–θ diffraction scans were carried out in the angular range from 10° to 30° 2θ at room temperature. The WAXS diffractograms were processed by MDI Jade software to calculate crystallinity.

Small Angle X-ray Scattering (SAXS). SAXS measurements were recorded at the Australian Synchrotron facility at a photon energy of 12 keV and two sample-to-detector distances of 1.020 and 7.160 m to give a q range of 0.002 to 0.2 $Å^{-1}$ after merging. Q is the scattering vector and is related to the scattering angle (2θ) and the photon wavelength (λ) by $q = 4\pi\sin(\theta)/\lambda$. Samples were loaded into 1.5 mm diameter quartz capillaries that were sealed with parafilm. The capillaries were held on a temperature-controlled mount with temperature control *via* a water bath connected to a brass block which is part of the sample holder. Temperatures up to 90 °C were reached and time was allowed for samples to equilibrate. The scattering from a blank (ethanol) was measured and subtracted for each measurement and temperature and data were normalized for total transmitted flux using a quantitative beamstop detector. The two-dimensional SAXS images were converted into one-dimensional SAXS profile (*I versus q*) by circular averaging using ScatterBrain, the SAXS software developed at the Australian Synchrotron. NCNR data Analysis IGOR PRO software and Primus were used to plot and analyze SAXS data.

Computation of LogP values. After constructing hexameric oligomers (See Figure 1) to represent the hydrophobicity of the parent polymer, the oligomers were minimized with the MM2 forcefield in Chem3D Pro version 13.0.2.3021. Then, octanol-water partition coefficient ($LogP_{\mathrm{oct}}$) were calculated with the chemical properties module. The Connolly surface area was calculated with a probe of 1.4 Å. $LogP_{\mathrm{oct}}$ values for the diblock copolymers were calculated with Materials Studio after Forcite geometry optimization of diblock copolymers using a 1.4 Å probe for the Connolly SA calculation.

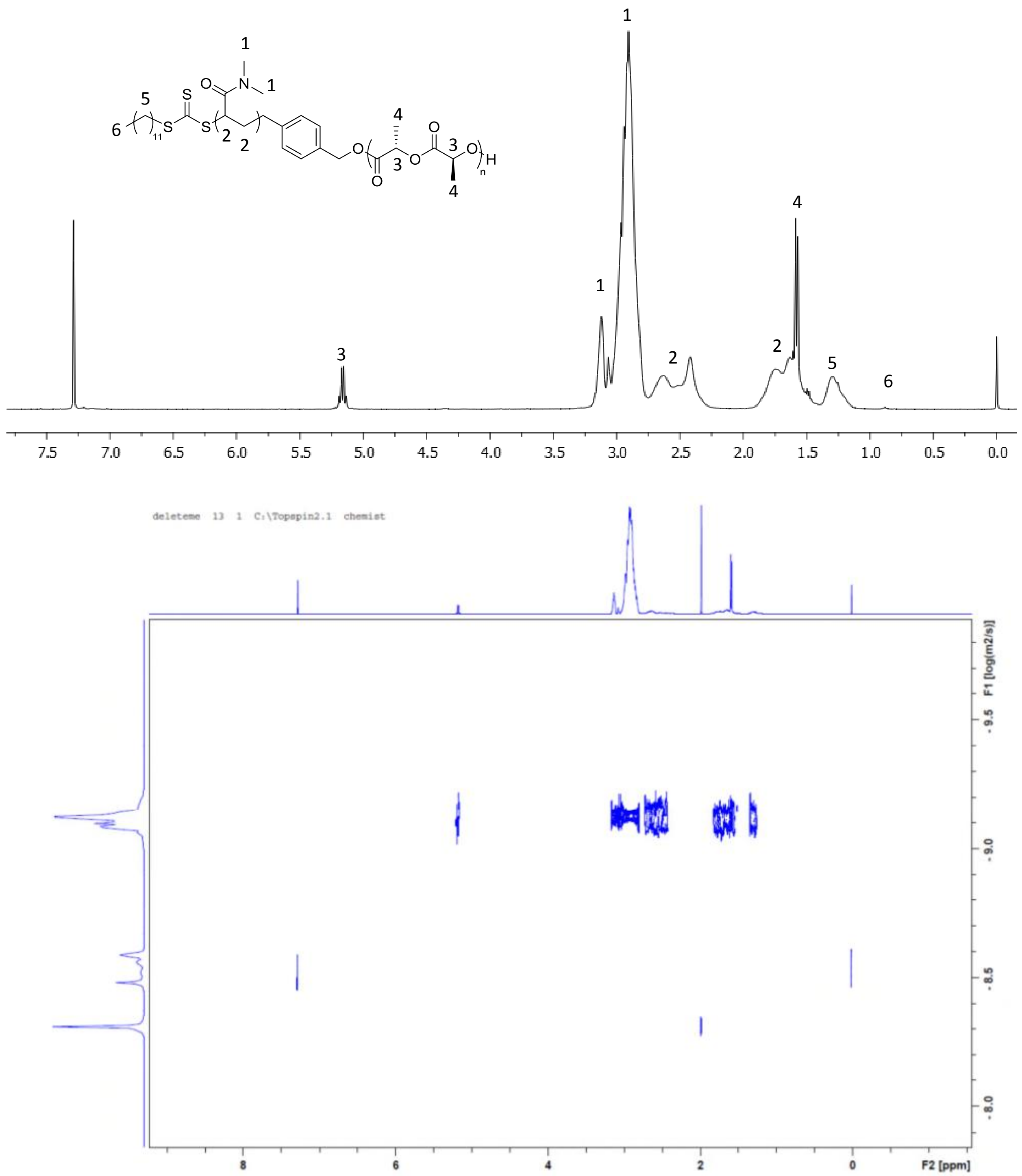

Figure S1. ^{1}H NMR spectrum (top) and DOSY (bottom) (400 MHz, $CDCl_3$) of PDMA-*b*-PLLA.

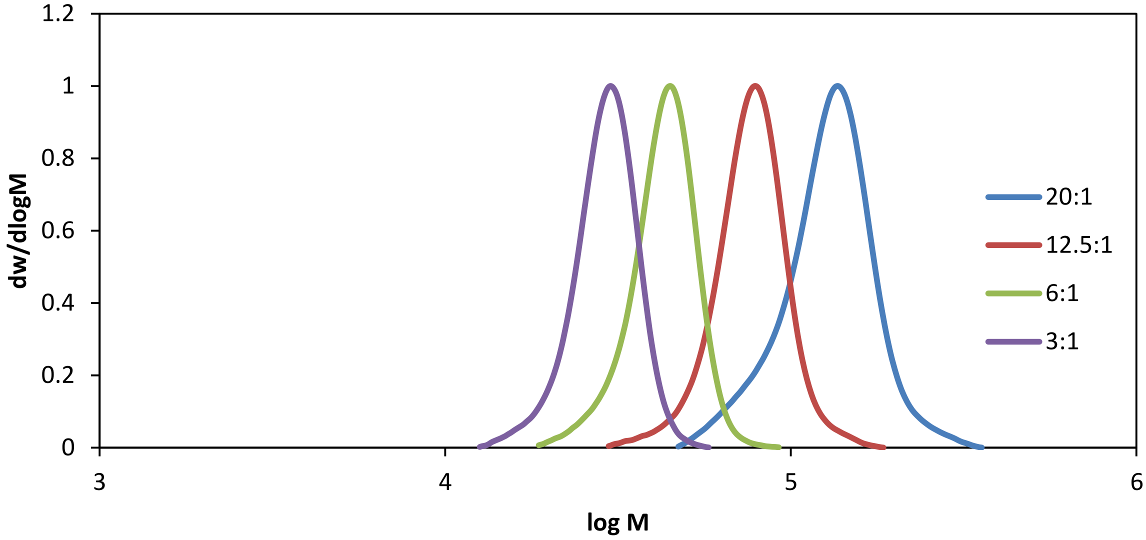

Figure S2. Overlaid refractive index and ultraviolet (λ = 309 nm) SEC chromatograms for PDMA-*b*-PLLA$_{48}$ diblock copolymers of corona-core ratios 20:1 ($Đ_M$ = 1.10), 12.5:1 ($Đ_M$ = 1.06), 5:1($Đ_M$ = 1.05) and 3:1 ($Đ_M$ = 1.05).

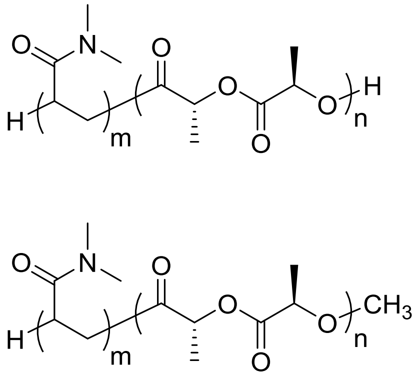

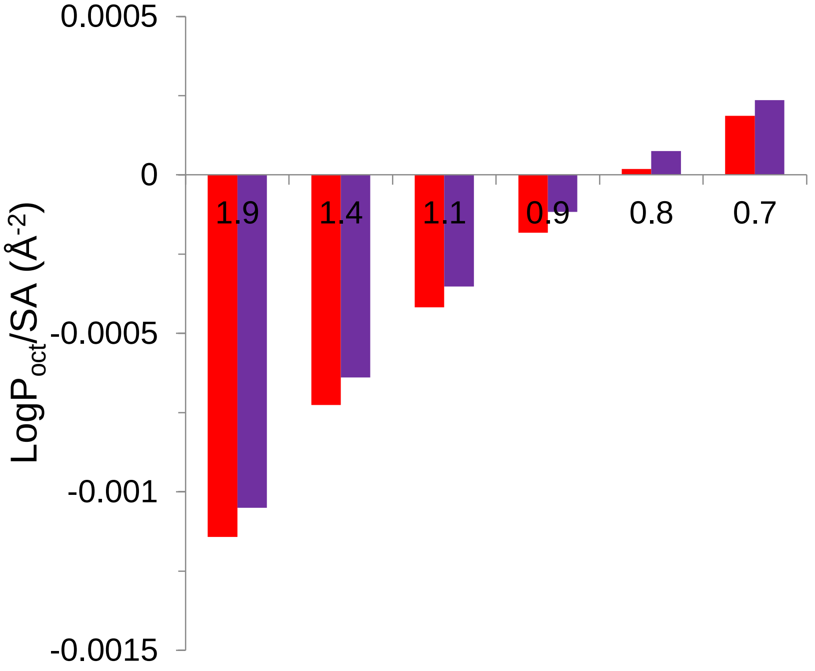

Figure S3. Octanol-water partition coefficients (*LogP*$_{oct}$) normalized by surface area (SA) (bottom) for PDMA-*b*-PLLA diblock copolymers (top). The x-axis shows the ratio of DMA:lactide units in the PDMA-*b*-PLLA diblock copolymer with OH endgroup (red columns) and MeO endgroup (purple columns). All the molecular models contained a constant number of DMA units (21) with varying numbers of lactide units. Calculations performed with Materials Studio after Forcite geometry optimization of diblock copolymers using a 1.4 Å probe for the Connolly SA calculation

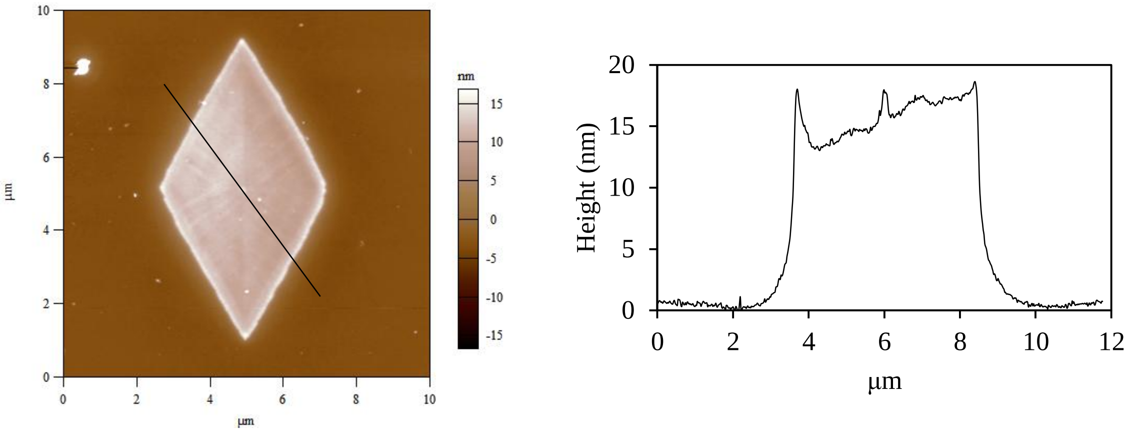

Figure S4. AFM and height profile of 20:1 $PDMA_{1000}$-*b*-$PLLA_{48}$ diamond platelet. Samples were self-assembled in ethanol at 90 °C for 8 h with subsequent slow cooling.

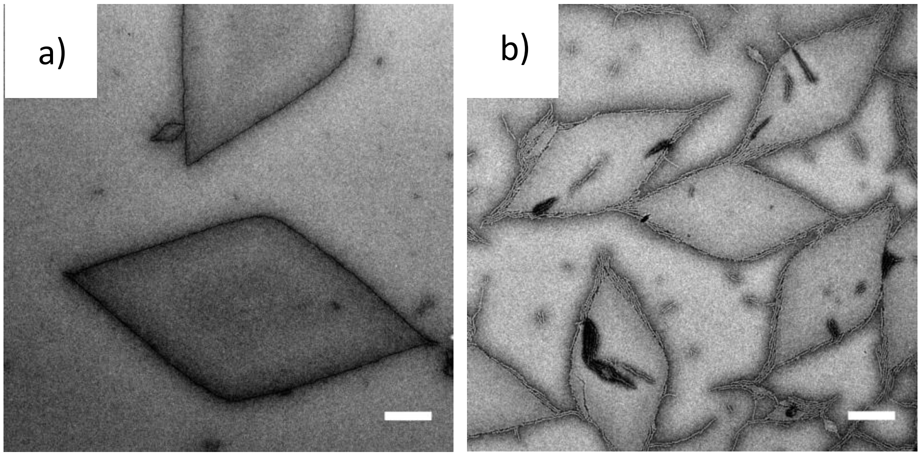

Figure S5. TEM micrographs of PDMA-*b*-$PLLA_{48}$ block copolymers of corona-core ratios of (a) 5:1, and (b) 3:1. Samples were self-assembled in ethanol at 90 °C for 18 h and cooled to room temperature. All samples were stained with uranyl acetate. Scale bar = 1 µm.

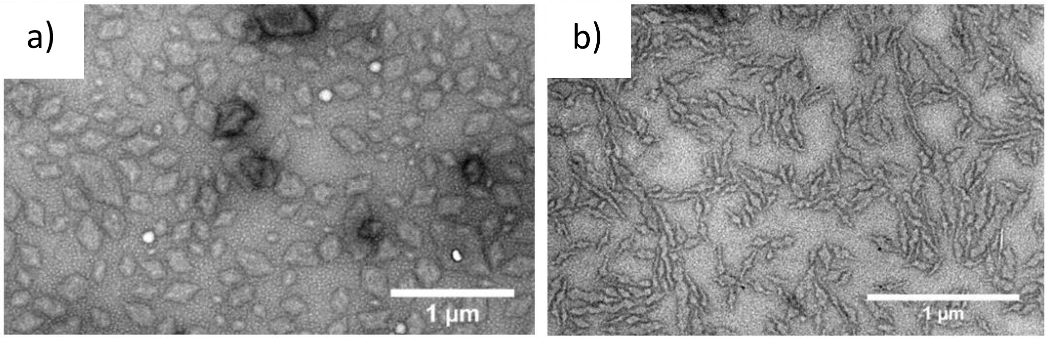

Figure S6. TEM micrographs of a series of PDMA-*b*-$PLLA_{25}$ block copolymers of corona-core ratios of (a) 10:1, (b) 5:1. Samples were self-assembled in ethanol at 65 °C for 18 h and cooled to room temperature. All samples were stained with uranyl acetate. Scale bar = 1 µm.

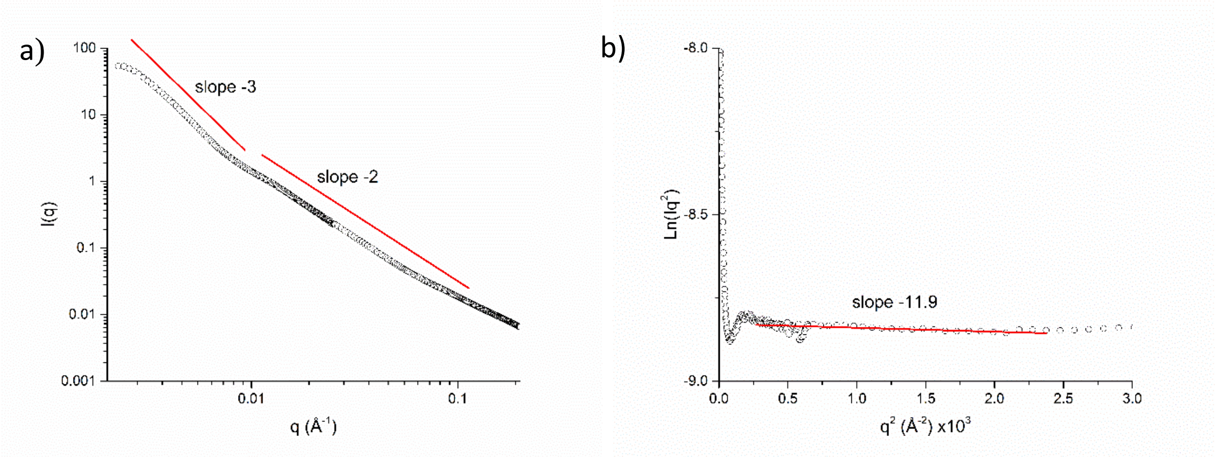

Figure S7. (a) Small-angle X-ray scattering profile for $PDMA_{1000}$-*b*-$PLLA_{48}$ (20:1 corona-core ratio) self-assembled at 65 °C in ethanol and (b) Guinier plot for platelet objects.

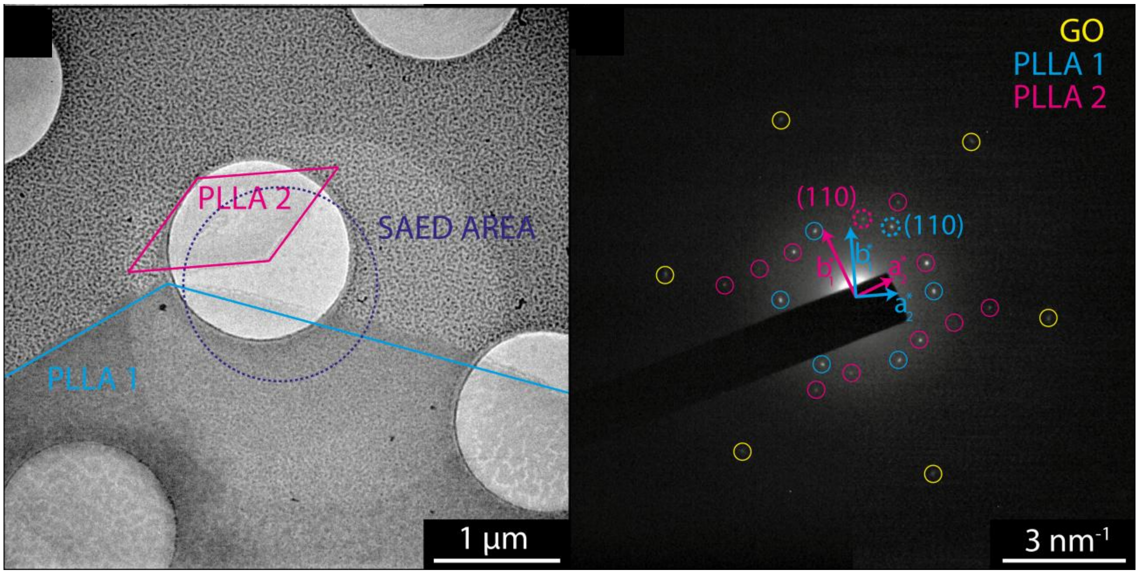

Figure S8. SAED analysis of PDMA$_{1000}$-*b*-PLLA$_{48}$ (20:1 corona-core ratio) diamond platelets.

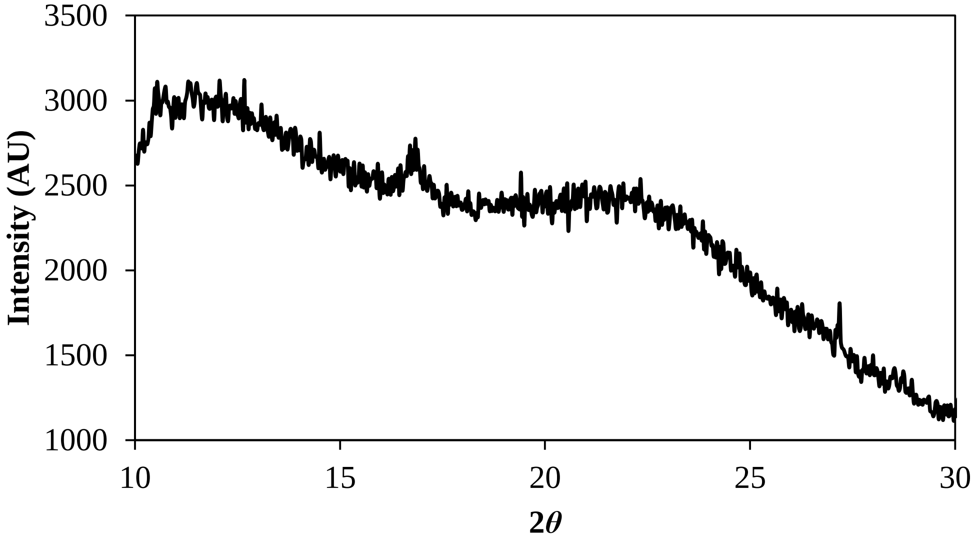

Figure S9. WAXS diffractogram of PDMA$_{1000}$-*b*-PLLA$_{48}$ (20:1 corona-core ratio) diamond platelets showing the 2θ peak at 16° characteristic of crystalline PLLA. The broad background signal is attributable to the amorphous large PDMA block for this block copolymer (20:1).

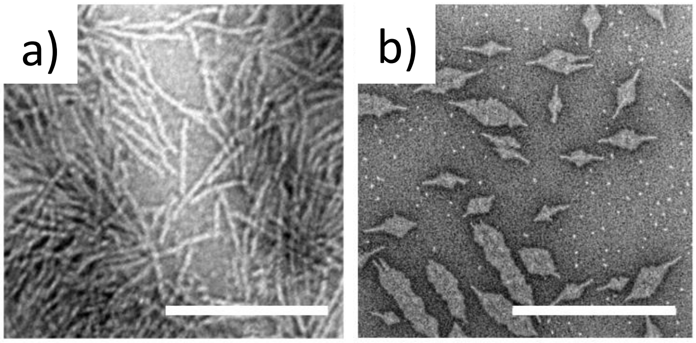

Figure S10. TEM micrographs of cylinder-forming (corona-core ratio 3:1) $PDMA_{150}$-*b*-$PLLA_{48}$ block copolymers (a) unmodified and (b) modified with a carboxylic acid group. Samples were self-assembled in ethanol at 90 °C for 8 h with and cooled to room temperature. Samples were stained with uranyl acetate. Scale bar = 1 µm.

a)

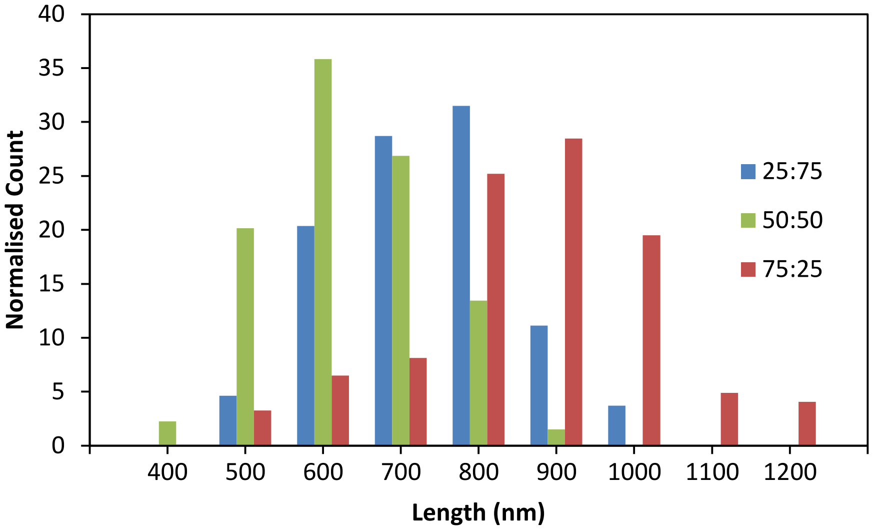

b)

Blending Ratio	L_w[a] (nm)	L_n[a] (nm)	L_w/L_n
25:75	754	735	1.03
50:50	734	695	1.06
75:25	965	912	1.06

[a] As determined by TEM analysis, see Materials and Methods section for details

Figure S11. (a) Histograms and (b) characterization data showing the length distribution of attached cylindrical micelles within hierarchical structures (prepared *via* PDMA-b-$PLLA_{48}$ blends of block ratios 20:1 and 3:1, at blending ratios of 25:75, 50:50 and 75:25, self-assembled in ethanol at 90 °C for 8 h and cooled to room temperature).

References

1 B. G. Lohmeijer, R. C. Pratt, F. Leibfarth, J. W. Logan, D. A. Long, A. P. Dove, F. Nederberg, J. Choi, C. Wade and R. M. Waymouth, *Macromolecules*, 2006, **39**, 8574-8583.

2 J. Skey and R. K. O'Reilly, *Chem. Commun.*, 2008, 4183-4185.

3 N. Petzetakis, A. P. Dove and R. K. O'Reilly, *Chem. Sci.*, 2011, **2**, 955-960.

4 A. Pitto-Barry, N. Kirby, A. P. Dove and R. K. O'Reilly, *Polym. Chem.*, 2014, **5**, 1427-1436.

5 J. P. Patterson, A. M. Sanchez, N. Petzetakis, T. P. Smart, T. H. Epps III, I. Portman, N. R. Wilson and R. K. O'Reilly, *Soft Matter*, 2012, **8**, 3322-3328.
